# Long-term outcomes of active surveillance after DOTATATE PET/MRI–confirmed gross total resection in WHO grade 2 meningioma

**DOI:** 10.1093/noajnl/vdaf210

**Published:** 2025-09-18

**Authors:** Jana Ivanidze, Kellen Vo Vu, Umberto Tosi, Se Jung Chris Chang, Kate Rosen, Hannah G Otis, Peter Chernek, Alexis Watson, Arsalan Haghdel, Valentina Marulanda Corzo, Sean H Kim, David Pisapia, Rajiv S Magge, Peter C Pan, Susan C Pannullo, Michelle Roytman, Eaton Lin, Sadek Nehmeh, Nicolas Karakatsanis, Arindam RoyChoudhury, Joseph R Osborne, Andrew Brandmaier, Kathryn Beal, Martin Zonenshayn, Rohan Ramakrishna, Philip Stieg, Benjamin Liechty, Jonathan P S Knisely, Theodore H Schwartz

**Affiliations:** Department of Radiology, Weill Cornell Medicine, New York, New York; MD Program, Weill Cornell Medicine, New York, New York; Department of Neurological Surgery, Weill Cornell Medicine, New York, New York; Department of Radiology, Weill Cornell Medicine, New York, New York; Department of Neurological Surgery, Weill Cornell Medicine, New York, New York; MD Program, Weill Cornell Medicine, New York, New York; Department of Pathology, Weill Cornell Medicine, New York, New York; Department of Radiology, Weill Cornell Medicine, New York, New York; Department of Radiology, Weill Cornell Medicine, New York, New York; Department of Radiology, Weill Cornell Medicine, New York, New York; Department of Radiology, Weill Cornell Medicine, New York, New York; Department of Pathology, Weill Cornell Medicine, New York, New York; Department of Neurology, Weill Cornell Medicine, New York, New York; Department of Neurology, Columbia University, New York, New York; Department of Neurological Surgery, Weill Cornell Medicine, New York, New York; Department of Radiology, Weill Cornell Medicine, New York, New York; Department of Radiology, Weill Cornell Medicine, New York, New York; Department of Radiology, Weill Cornell Medicine, New York, New York; Department of Radiology, Weill Cornell Medicine, New York, New York; Department of Population Health Sciences, Weill Cornell Medicine, New York, New York; Department of Radiology, Weill Cornell Medicine, New York, New York; Department of Radiation Oncology, Weill Cornell Medicine, New York, New York; Department of Radiation Oncology, Weill Cornell Medicine, New York, New York; Department of Neurological Surgery, Weill Cornell Medicine, New York, New York; Department of Neurological Surgery, Weill Cornell Medicine, New York, New York; Department of Neurological Surgery, Weill Cornell Medicine, New York, New York; Department of Pathology, Weill Cornell Medicine, New York, New York; Department of Radiation Oncology, Weill Cornell Medicine, New York, New York; Department of Neurosurgery, Icahn School of Medicine, Mt. Sinai Hospital, New York, New York

**Keywords:** DOTATATE, meningioma, PET/MRI, radiotherapy, somatostatin receptor 2

## Abstract

**Background:**

Somatostatin receptor 2 (SSTR2), a highly sensitive and specific meningioma biomarker, can be imaged with [68Ga]-DOTATATE PET, improving diagnosis and treatment. The role of postoperative radiotherapy (RT) for WHO grade 2 (WHO-2) meningiomas following gross total resection (GTR) remains controversial. We hypothesized that confirmation of GTR by DOTATATE PET/MRI followed by active surveillance would yield superior progression-free survival (PFS) compared to MRI-based GTR assessment alone.

**Methods:**

Patients with WHO-2 meningioma enrolled in a prospective registry were included if postoperative PET/MRI showed GTR and if they were managed with surveillance alone. All patients underwent serial MRI and/or PET/MRI follow-up. Kaplan–Meier analysis was used to determine PFS. A retrospective institutional comparator cohort of patients with WHO-2 meningiomas and MRI-determined GTR managed with surveillance alone was also evaluated.

**Results:**

Twenty-eight prospective subjects (61% women, mean age 61 years) met inclusion criteria. Meningiomas were located along the convexity (50%), falx (21%), and skull base (29%). Mean mitotic count was 5.1 per 10 high-power fields; mean follow-up was 28 months (range 5-64). In the PET/MRI cohort, PFS was 90.0% at 5 years. In comparison, the MRI-only cohort (*n* = 33) demonstrated a 5-year PFS of 67.0% (log-rank *P *= .04), despite similar clinicopathologic features.

**Conclusions:**

DOTATATE PET/MRI-confirmed GTR followed by active surveillance yielded significantly higher PFS compared to MRI-based GTR assessment in patients with WHO-2 meningioma. DOTATATE PET/MRI increases diagnostic certainty, enabling more accurate postoperative risk stratification and potentially avoiding unnecessary RT, supporting its integration into postoperative decision-making for WHO-2 meningioma.

Key Points

- For WHO-2 meningiomas, DOTATATE PET/MRI–confirmed GTR is associated with 90% 5-year progression-free survival
- PET/MRI-confirmed GTR is significantly better than MRI-based GTR at predicting recurrence
- These data support DOTATATE PET/MRI use to guide postoperative management

Importance of the Study

Postoperative management of WHO grade 2 meningiomas remains controversial, particularly regarding the role of adjuvant radiotherapy after gross total resection (GTR). This prospective study demonstrates that DOTATATE PET/MRI–confirmed GTR is associated with significantly improved long-term progression-free survival compared to conventional MRI-based assessment. These findings suggest that DOTATATE PET/MRI can more reliably identify patients who may safely forgo adjuvant radiotherapy, reducing overtreatment while preserving favorable outcomes. The results support integration of DOTATATE PET/MRI into postoperative risk stratification and decision-making in WHO-2 meningioma.

Meningiomas are the most common primary intracranial neoplasms, accounting for 38% of all primary central nervous system (CNS) tumors.^1^ Management of these mostly benign tumors comprises observation, surgery, or radiotherapy (RT). For symptomatic or progressively enlarging masses, surgery is often preferred, with the goal of maximal safe cytoreduction. If gross total resection (GTR) can be achieved, which includes removal of involved dura and bone, durable control and/or cure are possible.^2^ Unfortunately, GTR is not achieved in up to 50% of patients because meningiomas can encase or infiltrate adjacent neurovascular structures that must be preserved to avoid harm, leading to higher rates of recurrence and lower survival, necessitating subsequent active imaging surveillance for any residual or recurrent tumor.^3^

The current WHO grading system divides meningiomas into three grades, with higher grades having a significantly lower progression-free survival (PFS) and overall survival (OS).^4^ While observation and adjuvant irradiation are well established post-operative management approaches for GTR of WHO grade 1 (WHO-1) and 3 (WHO-3) meningiomas, respectively, optimal management of WHO-2 tumors remains controversial.^5^ In patients with WHO-2 meningiomas, postoperative RT is often pursued to mitigate recurrence risk.^6^ Several retrospective studies have shown that even after presumed GTR, RT is effective at increasing PFS for patients with WHO-2 meningiomas.^7–9^ However, the current paradigm for RT is fractionated treatment (fRT) of the entire resection bed (including a margin) over six or more weeks of daily treatment, exposing contiguous normal brain tissue to high doses of radiation, which has been associated with substantial adverse event rates (eg up to 19.6% overall and 14.3% persisting beyond 90 days in EORTC 22042-26042).^10^ More broadly, well-established adverse effects of RT for meningioma include dose-dependent alopecia,^11^ cognitive impairment,^12^ and pituitary dysfunction.^13^ Studies also show that some resected WHO-2 meningiomas never recur after resection alone, which raises the possibility that some completely resected WHO-2 meningiomas might be reasonably managed by observation alone.^14^ Thus, the optimal postoperative management of WHO-2 meningiomas with GTR remains undefined. This ambiguity is further reflected by two ongoing international phase III trials that are randomizing patients with WHO-2 meningiomas that are judged to have been completely resected on a post-operative MRI scan to either surveillance or early adjuvant fractionated irradiation to ascertain to what degree irradiation reduces the risk of tumor recurrence and whether the potential side-effects are justified.^15^^,^^16^

Extent of resection is an important prognostic factor in determining PFS for all meningiomas.^14^ The Simpson grade provides an intraoperative assessment of resection extent and relies on the surgeon’s subjective assessment of how much tumor and invaded adjacent bone had been removed.^17–19^ In the modern microsurgical era, recurrence rates for Simpson grades 1-3 are nearly indistinguishable.^20–22^ Moreover, postoperative MRI has been shown to be more sensitive for detecting residual tumor, compared with the surgeon’s intraoperative assessment, for predicting the risk of progression^23–26^^,^^27^ MRI-based assessment, although superior to intraoperative neurosurgical assessment, has limitations and cannot easily distinguish residual or recurrent tumor from post-treatment scarring and inflammation, with a reported sensitivity of 79% and specificity of 65% in distinguishing meningioma from tumor-free tissues.^28^^,^^29^ While contrast-enhanced MRI is routinely used to assess for residual tumor following meningioma resection, its sensitivity is limited in distinguishing true residual from postoperative dural enhancement. DOTATATE PET offers a functional assessment of somatostatin receptor expression that complements anatomical imaging, and prior studies have demonstrated its utility in identifying residual tumor not apparent on MRI.^30^ These limitations of MRI, along with the clinical importance of identifying residual disease, motivated the integrated imaging approach described in this study.

MRI also has anatomic limitations, having been shown to inadequately identify small lesions, infiltrative or “*en plaque*” lesions, osseous or parenchymal invasion, and tumor in deep locations such as the skull base and cavernous sinus.^29^^,^^31–33^ The limitations of conventional imaging in reliably determining resection extent and recurrence risk further highlights the need for objective, reproducible imaging biomarkers to guide postoperative decision-making.

[68Ga]-DOTATATE, a positron emission tomography (PET) radiotracer targeting somatostatin receptor 2 (SSTR2), has demonstrated superior clinical utility compared to MRI alone in identifying residual meningioma after surgery.^28^^,^^31–34^ Recent data also support its prognostic relevance: a 2023 study by Teske et al showed that postoperative DOTATATE PET positivity in WHO grade 1 meningiomas was associated with increased recurrence risk, suggesting utility for postoperative risk stratification even among lower-grade tumors.^35^

Nevertheless, there remains a knowledge gap regarding the effect of [68Ga]-DOTATATE PET/MRI-guided clinical decision making on clinical outcomes. In this study, we investigated whether using [68Ga]-DOTATATE PET/MRI to determine GTR after surgery was associated with improved PFS in patients with WHO-2 meningiomas. We hypothesized that patients with WHO-2 meningiomas and a GTR, as determined by negative postoperative [68Ga]-DOTATATE PET/MRI, would have a higher PFS compared with both our institutional retrospective comparison cohort in which GTR was determined by MRI alone, as well as previously published prospective^36^ and retrospective^7^ studies. We further propose that [68Ga]-DOTATATE PET/MRI can guide postoperative treatment decisions by reliably identifying patients for whom radiotherapy may be safely omitted.

## Materials and Methods

### Study Design and Participants

Following informed consent, patients were prospectively enrolled in our observational cohort study, in compliance with the Health Insurance Portability and Accountability Act and approved by our institutional review board. Inclusion criteria were age over 18, negative urine pregnancy test in female patients who were of reproductive age at the time of enrollment, GTR confirmed on postoperative MRI, and a histologic diagnosis of grade 2 meningioma based on 2021 CNS WHO criteria,^4^ including the presence of 4-19 mitotic figures per ten high-power fields, brain invasion, architectural features such as clear cell and chordoid architecture, or at least 3 atypical histological features (hypercellularity, necrosis, macronucleoli, patternless growth, and small-cell change). Exclusion criteria were histologic or molecular diagnosis of WHO-1or WHO-3 meningioma, prior history of cranial irradiation, other comorbidities with a poor prognosis that would significantly affect overall survival to a degree likely to preclude evaluation of PFS, and patient contraindications to gadolinium contrast or 3T MRI. Patients were also excluded if their first postoperative [68Ga]- DOTATATE PET/MRI or PET/CT showed any evidence of residual tumor. Following enrollment, subjects were imaged with [68Ga]-DOTATATE PET/MRI with a median interval of 4 months after surgery to allow for resolution of postoperative inflammation and improve specificity. Patients with findings of GTR by [68Ga]-DOTATATE PET/MRI at the time of baseline postoperative imaging were subsequently managed with active surveillance, including standard of care MRI every 3-6 months. Response Assessment in Neuro-Oncology working group (RANO) criteria^37^ were applied to determine progression, as detailed below.

### Procedures

Patients underwent gadolinium-enhanced MRI of the brain on a 3 Tesla clinical scanner (Biograph mMR, Siemens Healthineers, Erlangen), per institutional protocol, including 3‐D T1 SPACE (TR/TE: 600‐700 ms/11‐19 ms, 120-degree flip, 1 mm slice thickness) as well as 3‐D T2 FLAIR (TR/TE: 6300‐8500 ms/394‐446 ms, 120-degree flip, 1 mm slice thickness).

DOTATATE PET/MRI was performed approximately 6-12 months postoperatively in patients whose immediate postoperative MRI favored gross total resection and whose pathology confirmed WHO grade 2 meningioma. Imaging was evaluated in an integrated manner at the time of PET/MRI acquisition. PET acquisition was performed on the Siemens Biograph mMR scanner (Siemens Healthineers, Erlangen) in the majority (25/28 = 89%) of patients who underwent [68Ga]-DOTATATE PET imaging based on our previously published methodology,^28^^,^^38^ in dynamic 3D list-mode for a total of 60 min starting simultaneously with [^68^Ga]-DOTATATE injection and concurrent with the above-described MR acquisition. In a small subset of patients (3/28 = 11%), PET/CT was performed instead, on the Siemens Biograph mCT scanner (Siemens Healthineers, Erlangen), with PET acquisition parameters aligned to those of the PET/MRI protocol. In this subset of patients, contemporaneous MRI was obtained within 1 month of the PET/CT, and postcontrast 3D T1-weighted sequence obtained as part of that MRI was fused with the attenuation-corrected PET series using syngo.via MM Oncology workflow (Siemens Healthineers, Erlangen).

Absolute maximum standardized uptake values (SUV) were extracted for each queried lesion (eg postoperative cavity and associated enhancement), as well as SUV ratio referencing superior sagittal sinus (SUVR_SSS_), serving as background blood pool for normalization purposes based on our previously published methodology.^28^^,^^38^ As previously published, a SUVR_SSS_ of 3 was used as a positivity threshold to classify a PET-avid focus as meningioma.^39^ In this study, gross total resection (GTR) was defined based on the absence of residual [^68^Ga]-DOTATATE avidity on postoperative PET/MRI. This imaging-based definition was used irrespective of surgical Simpson grade, and included cases where involved bone and/or dura were not resected if no residual avidity was identified.

Pathology evaluation of resected tumor specimen included histomorphologic analysis with determination of WHO grade as per standard of care. In a subset of patients, SSTR2 immunohistochemistry (IHC) staining was additionally performed and quantified using a scale of I-V.

All patients were presented in a multidisciplinary brain tumor conference for review of pathology, operative and hospital course, imaging, and discussion of management.

### Outcomes

Radiographic progression was evaluated using RANO criteria^37^ at baseline postoperative PET/MRI and at each standard-of-care (SOC) follow-up MRI time point. Progression was defined as the appearance of a new enhancing lesion or unequivocal increase in size of a previously resected lesion, based on two-dimensional perpendicular measurements per RANO guidelines.^37^ Clinical progression was also reviewed, but all PFS events were radiographically defined.

### Retrospective Institutional Comparator Cohort

To contextualize findings from our prospective [68Ga]-DOTATATE PET/MRI cohort, we identified a retrospective institutional comparator cohort of patients with WHO-2 meningioma who underwent resection with MRI-determined GTR and were subsequently managed with active surveillance using MRI alone. These patients were treated between 2004 and 2019, prior to the routine implementation of [68Ga]-DOTATATE PET/MRI at our institution for postoperative assessment and before initiation of our prospective registry protocol. Eligibility criteria for this cohort included histologic confirmation of WHO-2 meningioma using contemporaneous histopathological criteria, postoperative MRI interpreted as showing GTR, and absence of adjuvant radiotherapy. Patients were excluded if postoperative MRI demonstrated residual tumor, if PET imaging was obtained, or if adjuvant RT was administered. Clinical, demographic, and histopathologic characteristics were collected through retrospective chart review. Recurrence events were identified through chart review and MRI follow-up reports. PFS was defined and analyzed in the same manner as in the prospective cohort.

### Statistical Analyses

Kaplan-Meier survival analysis was performed using the *lifelines* Python package.^40^ A log-rank test was used to compare our PFS curve to retrospective historical data (Pan et al).^7^ A one proportion z-test was conducted to compare our PFS results to published prospective historical data (Aghi et al).^36^

We performed Kaplan-Meier survival analysis for the retrospective institutional cohort analogous to our prospective PET/MRI-determined cohort and performed a log-rank test to compare PFS curves. To compare baseline characteristics between the prospective and retrospective institutional cohorts, we evaluated preoperative tumor size, tumor location (categorized as convexity, falcine, or skull base), number of mitoses per 10 high-power fields, Ki-67 index, and the presence of brain invasion, chordoid features, and clear cell histology (each analyzed using the Mann-Whitney U-test, chi-square, or Fisher’s exact test, as appropriate).

## Results

### Clinical and Demographic Characteristics of the Study Population

Twenty-eight patients met inclusion criteria for the prospective cohort. 17/28 subjects (61%) were women; mean age at the time of surgery was 58 years. Demographic characteristics of the cohort are summarized in Table 1. Mean preoperative 2-dimensional tumor size product by RANO criteria was 10.8 cm^2^ (range 1.71-33.6 cm^2^).

**Table 1.** Clinical, radiographic, and histopathologic characteristics of the prospective DOTATATE PET/MRI cohort and retrospective MRI-only comparator cohort

	PET-determined GTR cohort (*n* = 28)	MRI-determined GTR cohort (*n* = 33)	*P*-value
Age at time of surgery (years), mean	57.7 (range: 26.1-86.0)	57.6 (range: 20.0-96.0)	.9827 (Mann–Whitney)
Sex, *n* (%)			1.0000 (Fisher)
Female	17 (60.7%)	19 (57.6%)	
Male	11 (39.3%)	14 (42.4%)	
Preoperative size product (cm²), mean	10.8 (range: 1.7-33.6)	15.5 (range: 0.8-35.8)	.0450 (Mann–Whitney)
Tumor location, *n* (%)			.5622 (Chi-squared)
Convexity	14 (50.0%)	12 (36.4%)	
Falcine	6 (21.4%)	9 (27.3%)	
Skull base	8 (28.6%)	12 (36.4%)	
Surgical approach, *n* (%)			
Craniotomy	26 (92.9%)	n/a	
Endonasal	1 (3.6%)	n/a	
Transorbital	1 (3.6%)	n/a	
Mitotic figures per 10 HPF, mean ± SD	5.1 ± 1.9	5.1 ± 3.0	.5060 (Mann–Whitney)
Ki67 (%), mean	7.3 (range: 2.0-20.0)	10.5 (range: 5.0-30.0)	.6732 (Mann–Whitney)
Brain invasion present, *n* (%)			.4367 (Fisher)
Yes	2 (7.1%)	5 (15.2%)	
No	26 (92.9%)	28 (84.8%)	
Time from surgery to first PET (months), mean	8.8 (range: 2.0-48.6)	n/a	
Postoperative PET type, *n* (%)			
PET/CT	3 (10.7%)	n/a	
PET/MRI	25 (89.3%)	n/a	
Follow-up period (months), mean	28.4 (range: 5.4-63.7)	57.2 (range: 0.1-169.6)	.1197 (Mann–Whitney)

Data are presented as mean (range) or *n* (%), as appropriate. Statistical tests included Mann–Whitney U for continuous variables and Fisher’s exact or Chi-squared test for categorical variables, as noted. n/a = not applicable; variable not available or not assessed in the retrospective MRI-only cohort.

Tumor location was as follows: 8/28 (29%) of patients had tumors centered in the skull base, 14/28 (50%) had tumors located along the convexity, and 6/28 (21%) had tumors along the falx. 26/28 (93%) of patients underwent craniotomy, while 1/28 (4%) underwent endoscopic endonasal resection, and 1/28 (4%) underwent transorbital resection. The resections in the 28 patients were performed by a total of 6 neurological surgeons; 15/28 (54%) of surgeries were performed by a single neurological surgeon (author THS) while the other 13/28 (46%) were performed by 5 other neurological surgeons.

**Figure 1.**
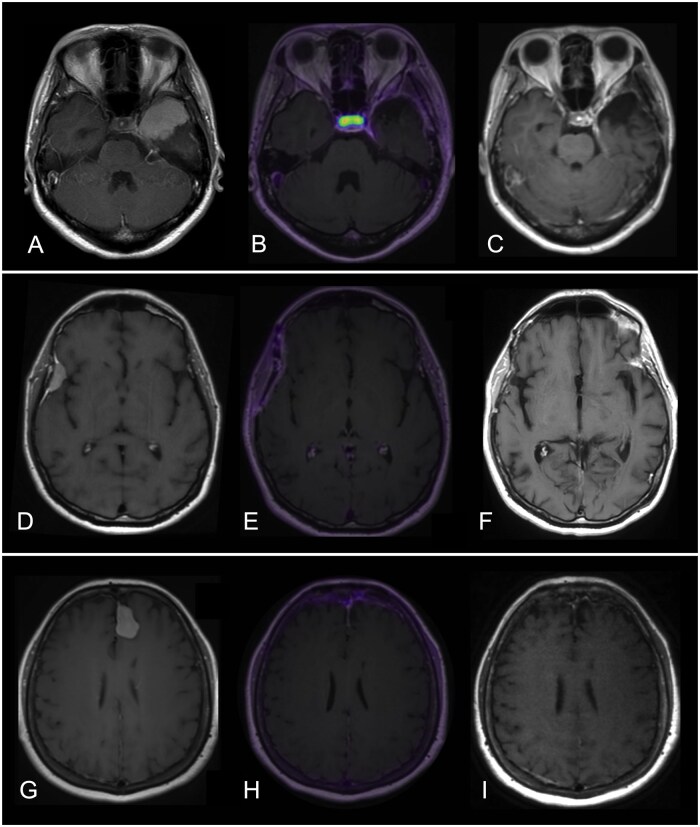
Three representative patients with WHO grade 2 meningiomas. A 36-year-old woman presented with headache, nausea, and blurry vision and was found to have an extra-axial dural-based homogeneously enhancing mass arising from the left greater sphenoid wing, measuring 4.2 × 4.0 cm with associated mass effect on the left medial frontal lobe (A). Pathology demonstrated CNS WHO grade 2 meningioma (6 mitoses per 10 high-power-fields, Ki-67: 8%). Post- operative follow-up MRI and DOTATATE PET/MRI (B) performed 3 months after resection demonstrated no evidence of residual or recurrent tumor. Smooth linear dural thickening and enhancement was visualized along the left anterior temporal convexity and left superior tentorial leaflet, with mild DOTATATE avidity below diagnostic threshold for meningioma (SUV 2.5, SUVRsss 1.7), favored to be reactive. Moderate physiologic avidity in the pituitary gland is consistent with expected SSTR2 expression. Serial MRI demonstrated interval decrease in linear dural thickening/enhancement, compatible with expected evolution of post-operative changes. Most recent follow-up MRI performed 3.8 years postoperatively (C) showed no evidence of residual or recurrent tumor. A 55-year-old man who presented with memory impairment was found to have an extra-axial dural-based homogeneously enhancing right frontotemporal convexity mass (D) which had demonstrated growth on serial MRI examinations, measuring 2.1 × 1.0 cm in the axial plane on preoperative MRI. Pathology demonstrated CNS WHO grade 2 meningioma (10 mitotic figures per 10 high-power-fields, Ki-67: 10%). Post- operative follow-up MRI and DOTATATE PET/MRI (E) performed 4 months after resection demonstrated no evidence of residual or recurrent meningioma; there was smooth linear dural thickening and enhancement subjacent to the craniotomy site, with mild DOTATATE avidity below diagnostic threshold for meningioma (SUV 3.0, SUVRsss 2.5), favored to be reactive. The most recent follow-up MRI performed 5.3 years postoperatively (F) demonstrates no evidence of residual or recurrent tumor. A 58-year-old woman who presented with memory loss was found to have an extra-axial dural-based homogeneously enhancing mass arising from the left aspect of the anterior falx cerebri, measuring 2.7 × 1.8 cm with associated mass effect on the left medial frontal lobe (G). Pathology demonstrated CNS WHO grade 2 meningioma (4 mitotic figures per 10 high-power-fields, Ki-67: 7%). Post- operative follow-up MRI and DOTATATE PET/MRI (H) performed 10 months after resection demonstrated no evidence of residual or recurrent meningioma; there was smooth linear dural thickening and enhancement along the left frontal convexity and anterior falx, surrounding the anterior third of the SSS without narrowing or occlusion. Corresponding DOTATATE avidity was mild and below diagnostic threshold for meningioma (SUV 2.7, SUVRsss 2.7), favored to be reactive. The most recent follow-up MRI performed 5.0 years postoperatively (I) showed no evidence of residual or recurrent tumor. PET/MRI fused images are displayed using the PET Rainbow color scale, windowed from 0 to 15 maximum SUV.

Three representative patients, including a patient with a skull base meningioma, a patient with a convexity meningioma, and a patient with a falcine meningioma, are shown in Figure 1.

### Histologic Characteristics of the Resected Tumors

All tumors met histologic criteria for WHO-2 meningioma. The mean mitotic count was 5.1 per 10 high-power fields (range, 4-11). In 3 of 28 patients (11%), mitotic activity was limited, and WHO-2 designation was assigned based on brain invasion (*n*  =  2) or chordoid morphology (*n*  =  1). SSTR2 immunohistochemistry (IHC) was performed in 5/28 cases (18%), all of which demonstrated strong membranous staining (IHC scores IV–V),^41^ consistent with high somatostatin receptor 2 expression.

### Progression-Free Survival

MRI follow-up data were available for a mean of 28 months (range 5-64 months). Kaplan-Meier analysis demonstrated the 30-month PFS to be 100%. At maximum follow up, our cohort demonstrated a 5-year PFS of 90.0% at last follow-up. None of the patients passed away; overall survival (OS) was 100% at maximum follow up. The median follow-up was 1.72 years (based on reverse Kaplan–Meier analysis). The 1-year and 2-year PFS were 100.0% (95% CI 100.0-100.0%), the 3-year PFS was 90.0% (95% CI 47.3-98.5%), and the 5-year PFS was 90.0% (95% CI 47.3-98.5%). The median PFS was not reached.

### Comparison with Retrospective Institutional Cohort

**Figure 2.**
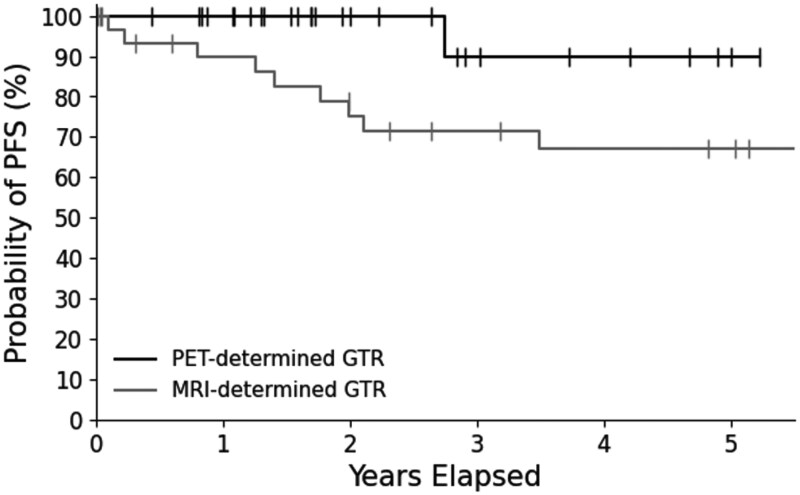
Progression-free survival (PFS) in WHO grade 2 meningioma with PET/MRI-confirmed gross total resection managed by surveillance. PFS in the PET/MRI cohort was 100% at 2.5 years, with a single recurrence at 33 months. The 5-year PFS was 90.0%. In the retrospective institutional cohort with MRI-determined GTR, 5-year PFS was 67.0%. Log-rank test comparing the two survival curves yielded a p-value of 0.04.

We identified a retrospective cohort of 33 patients with WHO-2 meningiomas treated with surgery alone from an IRB-approved retrospective registry of consecutive patients with meningiomas managed at our institution between 2004 and 2019. All patients in this cohort had a GTR as determined by postoperative MRI, were managed with MRI-based surveillance alone without DOTATATE PET imaging, and no patient received post-operative RT. Compared with the prospective PET/MRI cohort, tumor size was significantly larger in the retrospective group (median preoperative size product: 15.5 cm^2^ vs 10.8 cm^2^, *P* = .045; Table 1). All other clinical, radiographic, and histopathologic characteristics were similar between cohorts (Table 1). The median follow-up in the retrospective MRI-determined cohort was 57.2 months (Table 1). Kaplan–Meier analysis demonstrated a 5-year PFS of 67.0%, significantly lower than the 90.0% 5-year PFS observed in our prospective PET/MRI cohort (log-rank *P *= .04, Figure 2).

### Comparison with Published Literature

We then performed formal statistical comparisons with published benchmarks derived from large prospective and retrospective MRI-imaged cohorts. Compared to the prospective study by Aghi et al, which reported a 5-year PFS of 59%,^36^ our [68Ga]-DOTATATE PET/MRI cohort observed 5-year PFS was significantly higher (one-proportion z test, *P *< .001). We also compared our full Kaplan–Meier survival curve to that of Pan et al, who reported a 3-year PFS of 63%;^7^ this comparison yielded a significant difference in favor of our [68Ga]-DOTATATE PET/MRI cohort (log-rank test, *P *= .01). Comparison of PFS outcomes in our prospective cohort (PET/MRI-ascertained GTR) with these published benchmarks is summarized in Table 2. Together, these results suggest that [68Ga]-DOTATATE PET/MRI-determined GTR may be associated with improved PFS relative to historical MRI-based GTR determinations.

**Table 2.** Comparison of PFS outcomes in our prospective cohort (PET/MRI-Ascertained GTR) with published MRI-based GTR benchmarks

Study	Cohort type	Reported PFS	Corresponding prospective DOTATATE PET/MRI cohort PFS	Statistical comparison method and results
Aghi et al^36^	Prospective	5-year PFS: 59%	5-year PFS: 90%	One-proportion z test, *P* < .001
Pan et al^7^	Retrospective	3-year PFS: 63%	3-year PFS: 90%	Log-rank test, *P* = .01

### Clinical Recurrence and Subsequent Management

**Figure 3.**
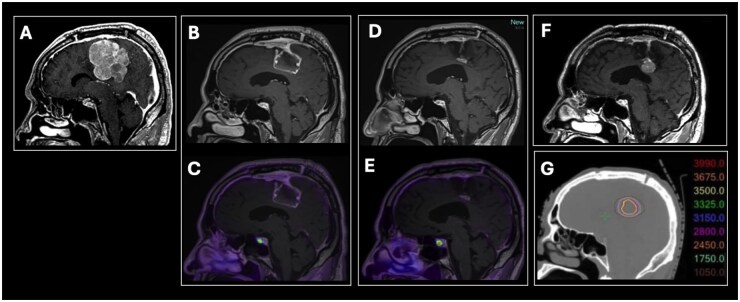
Single clinical recurrence in the prospective cohort. An 85-year-old man who presented with new onset left lower extremity weakness. Postcontrast sagittal T1-weighted MRI demonstrated a lobulated, heterogeneously enhancing right parafalcine dural-based mass (A). Pathology demonstrated CNS WHO2 meningioma (11 mitotic figures per 10 high-power-fields, Ki-67: 15%). Post- operative follow-up MRI (B) and DOTATATE PET/MRI (C) performed 2 months after resection demonstrated no evidence of residual or recurrent meningioma; there was smooth linear enhancement along the resection cavity margins, with mild corresponding DOTATATE avidity below diagnostic threshold for meningioma (SUV 2.2, SUVRsss 2.2), compatible with postsurgical change. A second MRI and PET/MRI performed 15 months post-operatively (D, E) showed expected interval contraction of the resection cavity with persistent mild DOTATATE avidity below diagnostic threshold for meningioma (SUV 2.2, SUVRsss 2.4) again compatible with postsurgical change, with no evidence of residual or recurrent meningioma. The patient subsequently did not return for surveillance MRI until 33 months postoperatively, at which time a new enhancing dural based mass was detected along the right aspect of the posteroinferior falx, at the site of the previously resected tumor, compatible with recurrence (F). The patient subsequently underwent salvage stereotactic radiosurgery with no immediate postprocedural complications. Figure (G) demonstrates isodose lines on the 5-fraction salvage linear accelerator radiosurgical salvage on non-contrast CT simulation sagittal image matching the postcontrast sagittal T1-weighted image shown in (F). PET/MRI fused images are displayed using the PET Rainbow color scale, windowed from 0 to 15 maximum SUV.

In our [68Ga]-DOTATATE PET/MRI cohort, a single recurrence was observed in an 86-year-old male patient who initially presented with new onset left lower extremity weakness progressive over several months. Imaging demonstrated a right parafalcine dural-based mass with imaging features suspicious for meningioma (Figure 3). The tumor had a preoperative size product of 17.3 cm^2^ by RANO criteria. The patient underwent a frontoparietal craniotomy with intraoperative assessment determining GTR. Postoperative [68Ga]-DOTATATE PET/MRI at 2 months post resection confirmed GTR. Pathology demonstrated WHO-2 meningioma, with 11 mitotic figures per 10 high-power-fields. Ki67-index was 15%. The recurrence, detected on follow-up surveillance MRI 33 months postoperatively, was identified as a 1.9 × 1.5 cm enhancing dural-based lesion along the right aspect of the posteroinferior falx. The patient received fractionated stereotactic radiosurgery (35 Gy in 5 fractions). However, the patient declined post-treatment clinical and imaging follow-up apart from his continuing to live independently (last assessed at 38 months following radiosurgical salvage).

## Discussion

In this study, we investigated whether [68Ga]-DOTATATE PET/MRI confirmation of GTR was associated with favorable PFS in patients with WHO-2 meningiomas managed without postoperative radiotherapy. We hypothesized that a negative [68Ga]-DOTATATE PET/MRI would provide better determination of the lack of residual tumor cells after surgery and predict increased PFS in the absence of fRT compared with MRI confirmation of GTR. Our findings demonstrate a PFS of 100% at 30 months and 90.0% at 5 years, with a recurrence in one patient (out of 28) who had a parafalcine meningioma. These results compare favorably with a variety of comparison groups with MRI-confirmed GTR, including our own institutional comparison (PFS of 67%), Aghi et al (NRG0539^5^; 3-year PFS of 59%),^36^ Pan et al (3-year PFS of 63%),^7^ and Lee et al (5- and 10-year PFS of 70% and 63%).^9^ Together, these data support the utility of [68Ga]-DOTATATE PET/MRI in identifying patients with GTR of WHO-2 meningiomas who may safely forgo adjuvant radiotherapy following surgery, thereby reducing overtreatment without compromising long-term outcomes.

Previous studies have demonstrated that [68Ga]-DOTATATE PET exhibits higher sensitivity than contrast-enhanced MRI for detecting meningiomas, with similar specificity, and has proven particularly effective in delineating tumor extent and distinguishing recurrence from treatment-related changes in the postoperative setting.^28^^,^^29^ While these studies have highlighted the diagnostic advantages of [68Ga]-DOTATATE PET and reported its utility in short-term postoperative management, they have not evaluated its role in guiding clinical decision-making or its impact on long-term outcomes. Our study addresses this gap by assessing the use of [68Ga]-DOTATATE PET/MRI to guide postoperative management, with follow-up data extending up to 60 months. The excellent PFS outcomes in our cohort suggest that postoperative PET/MRI can help identify patients with WHO-2 meningioma who may safely forgo routine radiotherapy, potentially reducing overtreatment while maintaining favorable outcomes. These findings support the integration of [68Ga]-DOTATATE PET/MRI into postoperative decision-making.

While multiple recent studies provide justification for incorporating DOTATATE PET/MRI in the postoperative management of meningiomas, they focused primarily on the scenario of incomplete resection and subsequent postoperative radiotherapy. A prospective registry study incorporating all WHO grades demonstrated safety and efficacy of DOTATATE PET/CT-guided radiotherapy.^42^ A separate recent prospective institutional cohort study, also incorporating all WHO grades, illustrated the utility of DOTATATE PET/MRI-guided radiosurgical treatment as well as response assessment.^30^ In that study, PFS was 83% at maximum follow-up, suggesting that salvage SRS to postoperative recurrence can be an excellent management approach in this population, as was the case in the single patient who recurred in our cohort.^30^ In WHO-3 meningiomas specifically, [68Ga]-DOTATATE PET/MRI may differentiate between *de novo* and secondary progressive disease, underscoring its potential value across the spectrum of meningioma care.^43^

Our study adds to this growing body of evidence by demonstrating a role for DOTATATE PET/MRI in the postoperative management of WHO-2 meningioma following PET/MRI-determined GTR. In contrast to prior studies focused on diagnosis^28^^,^^29^^,^^38^^,^^42^^,^^44^ and radiotherapy planning,^30^^,^^34^^,^^42^ here we investigated DOTATATE PET/MRI as a tool to guide patient selection for surveillance. In addition to comparisons with external benchmarks, we evaluated an internal retrospective cohort of patients with WHO-2 meningioma and MRI-determined GTR who did not undergo DOTATATE PET/MRI. This institutional cohort exhibited substantially lower progression-free survival despite similar surgical management, highlighting the limitations of conventional imaging and the potential of DOTATATE PET/MRI to improve postoperative risk stratification in WHO-2 disease. The improved outcomes observed in patients without residual avidity on DOTATATE PET/MRI suggest that PET may offer superior prognostic stratification compared to MRI alone, underscoring the value of future prospective studies examining patients with discordant MRI and PET findings.

While the current analysis focused on patients with concordant MRI- and PET-determined GTR managed without radiotherapy, patients with discordant findings—specifically, MRI-negative but DOTATATE PET-positive—represent an important clinical subgroup. Outcomes in this population are the subject of a separate ongoing investigation at our institution, which aims to compare local control rates in patients who underwent postoperative radiotherapy based on PET positivity despite MRI-suggested GTR. These data may further clarify the utility of DOTATATE PET in refining postoperative management algorithms for WHO-2 meningioma.

In WHO grade 1 meningiomas, a recent study by Teske et al reported that DOTATATE PET findings were associated with clinical outcomes, including PFS, underscoring the value of SSTR2-targeted imaging for postoperative prognostication across the spectrum of meningioma grades. While the current study differs in population and methodology, our results align with and extend these findings by focusing on a higher-risk grade 2 cohort, where clinical management decisions such as the omission of radiotherapy carry more uncertainty and potential impact.

Our study includes several limitations. There is some heterogeneity in the timing of postoperative PET/MRI relative to time of surgery, which may affect consistency of imaging interpretation. Furthermore, while we demonstrate follow-up for up to 5 years, the mean follow-up period is 28 months, which may limit the generalizability of our findings. Inflammatory cells express low levels of SSTR2,^45^ thus postoperative inflammation may result in a false-positive baseline PET/MRI examination, suggesting a lesion represents residual tumor when it is in fact postoperative reactive dural thickening/enhancement. This was observed in a prior [68Ga]-DOTATATE PET/CT study, which compared Simpson grade assessments to DOTATATE PET/CT findings in a cohort with varying WHO grades and found unexpected DOTATATE avid foci in 40.5% of cases presumed to have GTR by Simpson grade.^26^ However, Simpson grading has known limitations in accuracy,^23–26^ and the median time between surgery and PET/CT in that study was only 25 days—a time period during which postoperative inflammation is likely to persist, which may increase the likelihood of PET positivity due to postoperative inflammation. In contrast, postoperative baseline imaging in our cohort was performed at a mean time of 8.8 months after resection to allow for resolution of postoperative inflammation, and we additionally applied a previously validated SUVR_SSS_ diagnostic threshold.^20^^,^^39^ In our cohort, no patients demonstrated DOTATATE avidity above this diagnostic threshold, suggesting the absence of residual tumor.

Given the limitations of Simpson grade, postoperative MRI and [68Ga]-DOTATATE PET/MRI may provide a more reliable assessment of resection extent.^23–26^ While the term ‘gross total resection’ is often used in the context of Simpson grading, our definition is based on postoperative DOTATATE PET imaging. This distinction is important, as PET may detect residual disease not evident on surgical inspection or conventional MRI, and conversely, may confirm the absence of viable tumor even when resection of involved dura or bone is incomplete.

This study is additionally limited by the modest sample size and the lack of matched preoperative tumor volumes or locations between cohorts, which may introduce potential confounding. While tumor size and site are important prognostic features, the primary focus of this work is on the postoperative assessment of residual disease. Specifically, we demonstrate that DOTATATE PET imaging offers greater sensitivity than contrast-enhanced MRI alone for detecting subtle postoperative residual, which may more accurately inform recurrence risk and guide radiotherapy decisions. Additionally, multicenter validation of DOTATATE PET–guided postoperative assessment is needed to enable broader clinical adoption. Such efforts would ideally involve standardized imaging protocols, central radiology review, and harmonized histologic and molecular annotation across institutions.

SSTR2 expression has been reported in 100% of meningiomas in pathology studies.^46^ While rare, the possibility of SSTR2-negative (and therefore, PET-negative) meningioma must be considered.^47^ Given the low frequency of SSTR2-negative meningiomas, SSTR2 is not routinely included in standard-of-care pathologic evaluation of meningioma. A strong correlation between SSTR2 IHC and [68Ga]-DOTATATE PET SUV has previously been demonstrated in clinical cohorts,^29^ and thus SSTR2 IHC is not considered a prerequisite in studies incorporating DOTATATE PET. In our cohort, SSTR2 IHC was available in 17% of patients, and demonstrated strong positive staining in 100% of cases. Although DOTATATE-negative meningiomas are uncommon, this possibility underscores the importance of serial MRI follow-up, even after DOTATATE-negativity, which remains a standard of care and provides an essential safeguard for recurrence detection in patients managed with surveillance.

Emerging data suggest that molecular and epigenetic features—including CDKN2A deletion, TERT promoter mutation, copy number analyses, and methylation-based classification—will be important to integrate into risk stratification frameworks for meningioma.^48–53^ However, these assays are not yet universally available and were not routinely performed in our cohort. In the clinical setting, most WHO grade 2 tumors are still classified using histopathology alone, and we deliberately focused on the value of DOTATATE PET imaging in that context. The 2021 WHO Classification of CNS Tumors incorporates molecular criteria, including CDKN2A homozygous deletion and TERT promoter mutation, in the diagnosis of anaplastic (WHO grade 3) meningioma. By definition, these alterations were not present in the tumors included in our WHO grade 2 cohort. While molecular profiling is increasingly used to refine risk stratification in meningioma, its clinical implementation remains variable, and such testing was not uniformly performed in the earlier retrospective cohort. Nevertheless, the strong predictive value of PET-determined gross total resection in this study suggests that the absence of residual SSTR2-expressing tumor may supersede some molecular risk features in guiding postoperative management decisions. Future studies with larger prospective cohorts and integrated molecular profiling will be important to further explore these relationships. Future studies with larger cohorts and integrating PET with molecular profiling are warranted to validate these findings and identify additional predictors of recurrence.

While this study focused on patients with DOTATATE PET-confirmed GTR, the institutional and literature comparison cohorts included patients with GTR defined by MRI alone, introducing an inherent asymmetry in residual tumor assessment. However, this reflects current clinical practice and ongoing trials (eg EORTC-1308),^54^ which rely solely on MRI to determine GTR. Our findings suggest that DOTATATE PET may more accurately confirm true GTR and help refine postoperative management.

In this prospective cohort, DOTATATE PET/MRI was obtained approximately 6-12 months postoperatively in patients with immediate postoperative MRI favoring GTR and confirmed WHO grade 2 histology. As such, we did not include cases where MRI may have suggested GTR but PET demonstrated residual disease. Such patients were treated in a non-protocolized manner at our institution, with variable use of postoperative radiation versus active surveillance. Prior work has shown that DOTATATE PET can improve delineation of postoperative residual meningioma, with implications for treatment planning and follow-up strategies.^30^ Future investigations may help clarify the role of DOTATATE PET in refining postoperative management algorithms for patients with WHO grade 2 meningioma and PET/MRI discordance.

In our cohort, one patient experienced recurrence despite [68Ga]-DOTATATE PET/MRI-determined GTR. Notably, this patient had a falcine meningioma, a location that has historically demonstrated higher rates of recurrence compared to convexity lesions.^55^^,^^56^ Furthermore, the tumor was among the largest in the cohort, with one of the highest mitotic indices observed in our cohort. The recurrence was demonstrated on standard-of-care MRI and was successfully managed with salvage stereotactic radiosurgery. This case demonstrates that serial imaging can be an effective strategy for managing patients found to be GTR by DOTATATE PET/MRI postoperatively, avoiding the potential complications associated with radiotherapy for most patients with low recurrence risk, while still providing a pathway for effective intervention in the event of recurrence. An additional limitation of our study is the non-randomized comparison with a retrospective institutional cohort. Although the comparator group was drawn from the same institution and demonstrated no significant differences in tumor location or mitotic index, it was not explicitly matched to the prospective cohort, and residual confounding cannot be fully excluded. Notably, preoperative tumor size was significantly larger in the retrospective group. However, the magnitude of the observed difference in progression-free survival between cohorts was substantial, making it unlikely that this or other unmeasured confounders could entirely account for the effect. Additionally, WHO grading criteria for meningioma were revised in 2016 (adding brain invasion as a criterion for grade 2)^57^ and again in 2021 (incorporating molecular markers such as CDKN2A/B deletion and TERT promoter mutation),^4^ which may have introduced differences in diagnostic classification or management over time. Finally, although 3D T1-weighted postcontrast MRI has been standard institutional practice for over a decade, minor changes in MRI protocols or interpretive thresholds could have introduced variability in the assessment of GTR.

Taken together, our findings suggest that [68Ga]-DOTATATE PET/MRI can be used as a more sensitive biomarker for the detection of residual disease compared with conventional MRI alone and may inform the decision to safely omit postoperative radiotherapy. Continued standard-of-care MRI surveillance is recommended to identify any recurrence. Integration of [68Ga]-DOTATATE PET/MRI into postoperative management algorithms can help reduce RT overtreatment and improve long-term outcomes.

## Data Availability

De-identified data underlying the results reported in this article will be shared upon resonable request to the corresponding author.
